# Exploration of Leisure Constraints Perception in the Context of a Pandemic: An Empirical Study of the Macau Light Festival

**DOI:** 10.3389/fpsyg.2022.822208

**Published:** 2022-02-18

**Authors:** Xi Li, Jiamin Liu, Xinwei Su, Yinan Xiao, Changbin Xu

**Affiliations:** ^1^Faculty of International Tourism and Management, City University of Macau, Macao, Macao SAR, China; ^2^Liming Vocational University, Quanzhou, China; ^3^Tourism College, Beijing Union University, Beijing, China; ^4^Hainan College of Vocation and Technique, Hainan, China

**Keywords:** leisure constraints, festival, COVID-19 pandemic, Macau, perception difference

## Abstract

Individuals' capacity to participate in leisure activities is contingent upon their ability to overcome obstacles. It is worthwhile to investigate how individuals perceive constraints on their leisure activities participation during the COVID-19 pandemic. This study demonstrates that the connotation of leisure constraints during pandemic includes personal health concerns, shock on economic revenue, reduced freedom of travel, and inconvenience associated with epidemic prevention. Reduced travel freedom is the most influential factor on participation intentions, followed by personal health concerns. Significant differences in perceptions of constraints are observed between groups with different characteristics.

## Introduction

Academics and industry have long recognized leisure activities as critical components of effective personal well-being enhancement (Snape et al., 2017). Simultaneously, this type of positive impact on individuals can have a ripple effect on surrounding communities and society. As a result of the COVID-19 epidemic's rapid growth and spread, individuals have suffered intolerable psychological consequences (Xiao, 2020). Moreover, the COVID-19 pandemic has forced people to postpone or even cancel numerous leisure activities. This has a profound effect on the health and well-being of individuals and communities, posing a threat to their future viability (Jamal and Budke, 2020). The impact of the COVID-19 pandemic on people's leisure activities is primarily due to obvious restrictions on the amount of space available, the type of recreational activities available, and the scope of recreational activities available (Strauss, 2020). As a result, some scholars have noted that the leisure industry has emerged as one of the sectors hardest hit by the COVID-19 pandemic (Neuburger and Egger, 2021).

Existing research on leisure activities in the context of the COVID-19 pandemic is primarily focused on two aspects: the growth pattern of leisure activities in the aftermath of the pandemic and the leisure behavior of individuals during the pandemic. In terms of growth, scholars have primarily examined the detrimental effect of the COVID-19 pandemic on leisure activities (Mowatt, 2021). The research subjects for leisure behavior are relatively diverse: negative emotions, such as anxiety in the context of a pandemic (Kim and Kim, 2020); leisure activities for females in relatively enclosed spaces (Giles and Oncescu, 2021); and so forth. Given the magnitude and breadth of the impact, people's behavior during the COVID-19 pandemic may differ significantly from normal (Cori et al., 2020).

Leisure constraint is a subject that has garnered considerable attention from academics (Schneider and Wilhelm Stanis, 2007). Existing research on leisure constraints focuses primarily on the perceptions of various types of subjects in regular situations, such as company employees (Hubbard and Mannell, 2001), and patients with fibromyalgia syndrome (Loucks-Atkinson and Mannell, 2007). Additionally, there are studies that examine constraints associated with various types of leisure activities, including cruising (Hung and Petrick, 2012), fishing (Lyu and Oh, 2014), festivals (Boo et al., 2014), solo travel (Chung et al., 2017), and Silver-haired Traveling (Wen et al., 2020). There is a dearth of research on leisure constraints in times of public health crisis, such as the Covid-19 pandemic. While some scholars, such as Yang et al. (2011) examined the leisure constraints of undergraduate students in the aftermath of SARS, Jian et al. (2021) noted that scholars pay scant attention to leisure constraints during social emergencies such as epidemics, natural disasters, and global economic crises. Additionally, some scholars noted that, while the dimensions of intrapersonal, interpersonal, and structural constraints identified in the study prior to the COVID-19 outbreak remain relevant today, the COVID-19 outbreak appears to have resulted in the emergence of new constraints that will affect consumer behavior (Alexandris et al., 2021). Furthermore, it is discovered that scholars have not yet reached consensus on whether leisure constraints fall under the intrapersonal, interpersonal, and structural constraints classification (Ito et al., 2020).

In light of the aforementioned research gaps, this study has two primary objectives: first, to investigate the perception of leisure constraints caused by the Covid-19 pandemic; and second, to use a festival as a research context to investigate the differences in perceptions of leisure constraints among different groups. Simultaneously, it demonstrates a possible path for management to address leisure constraints, allowing people to engage in leisure activities while maintaining physical and mental health in the post-pandemic era.

## Literature Review

### The Concept and Composition of Leisure Constraints

While discussions of leisure constraints began in the early 1960s (Buchanan and Allen, 1985), it was not until the 1980s that a theoretical framework for leisure constraints was developed (Hung and Petrick, 2012). The connotation of leisure constraints has evolved, from the initial analysis of leisure-related impediments (Crawford and Godbey, 1987) to the development of a hierarchical model of leisure constraints (Crawford et al., 1991), which emphasizes the existence of hierarchical leisure constraints. Jackson et al. (1993) proposed that leisure constraints refer to factors that individuals perceive as impeding their participation in and enjoyment of leisure and entertainment activities.

For the connotation of leisure constraints, scholars have proposed a variety of models, including a two-dimensional conceptual model, a three-dimensional conceptual model, and even a seven-dimensional conceptual model. Jackson and Searle (1985) classified leisure constraints into two categories: internal (individual interest and capacity) and external (time, money and environment). Internal constraints refer to the psychological state and characteristics of an individual that influence their choice and participation in leisure activities. External constraints are factors that restrict people's participation in leisure activities, and they are frequently influenced by the external environment.

Crawford et al. (1991), for example, proposed a hierarchical constraint model that encompasses three distinct types of constraint: structural constraint, intrapersonal constraint, and interpersonal constraint. Since then, a large number of scholars have recognized and utilized this model (Hubbard and Mannell, 2001; Kerstetter et al., 2005; Loucks-Atkinson and Mannell, 2007). Structural constraints are imposed by the stages of the family life cycle, the family's financial resources, the seasons, the climate, and work schedules. Internal constraints include their perception of pressure, the attitudes of their reference group, prior socialization with specific leisure activities, and their self-perception of their ability to participate (Toogood et al., 2014). Locating suitable travel companions is an illustration of an interpersonal constraint (Crawford et al., 1991).

Nonetheless, some scholars appear to disagree with this conclusion. Alexandris and Carroll (1997), for example, categorize constraints into seven categories: individual/psychological, lack of knowledge, facilities/services, accessibility/financial, lack of interest, lack of partners, lack of time. Casper et al. (2011) emphasized that structural constraints relating to “inadequate time” are fundamentally distinct from structural constraints relating to “facilities” in terms of content. As can be seen, the specific composition of leisure constraints warrants additional investigation.

Over the last three decades, research on constraints has emerged as a central theme in leisure and tourism (Dale and Ritchie, 2020). This study summarizes the findings of previous research from three perspectives. To begin, from the perspective of the research subjects, marginalized groups have become a point of focus for scholars. For example, scholars have focused on the travel and leisure constraints faced by the elderly, females, and disabled groups (Khan et al., 2019; Wen et al., 2020). Second, scholars have conducted a series of studies on various types of leisure activities, including festivals, fishing, and cruises (Hung and Petrick, 2012; Boo et al., 2014; Lyu and Oh, 2014), in light of leisure constraints. Thirdly, the effect of cultural differences on leisure subjects' perceptions of leisure constraints has emerged as a major source of concern for scholars. For instance, Chen et al. (2013) discussed cultural differences in perceptions of leisure constraint.

However, because the aforementioned studies were all conducted in a regular social setting, there is a scarcity of research on leisure constraints in times of public health crisis, such as the Covid-19 pandemic. Due to the fact that one's perception of leisure constraints has an effect on one's participation in leisure activities (Crompton et al., 2005), it is necessary to investigate the constraints that leisure activity participants face during the pandemic era.

### Leisure Constraints and Participate Intention

Behavioral intention is frequently used to refer to an individual's anticipated or planned future behavior (Ajzen, 1991). The extent to which constraints affect behavioral intentions, on the other hand, remains debatable. According to some scholars, both individual and structural constraints will have a significant impact on tourists' intention to visit. For instance, in the context of wine tourism, leisure constraints have a significant negative effect on behavioral intentions (Bonn et al., 2016). Nyaupane and Andereck (2008) discovered that the most critical preconditions for the tourism decision-making process are structural constraints (time, cost, and accessibility). Simultaneously, travel restrictions have been shown to significantly reduce individuals' willingness to take vacations (Hung and Petrick, 2012). However, some researchers have discovered that perceptions of leisure constraints have little or no effect on behavioral intentions (Kay and Jackson, 1991; Zhang et al., 2012). This study will investigate the relationships described above in the context of the Covid19 pandemic.

### Disparities Between Groups in Their Perceptions of Leisure Constraints

By and large, different groups perceive leisure constraints differently (Shores et al., 2007; Godbey et al., 2010). The current body of knowledge regarding perception differences in leisure constraints is primarily concerned with groups that differ in terms of demographic and behavioral characteristics. Jun et al. (2009) discovered, for instance, that groups of varying ages and races have markedly different perceptions of leisure constraints. Bülent et al. (2010) established that perceptions of leisure constraints vary according to marital status and gender. Likewise, a study on festival attendance mentions similar findings (Boo et al., 2014). Kim et al. (2020) conducted a recent study in which they examined attitudes toward leisure sports participation among groups with varying demographic characteristics in the context of the pandemic. Leisure constraints are also perceived differently by groups with disparate social and behavioral characteristics, such as income and education (Jun et al., 2009; Bülent et al., 2010), leisure motivation, and so on. Chang et al. (2011) and Boo et al. (2014), for e.g., both noted that individuals with lower incomes face greater intrapersonal, interpersonal, and structural constraints during their leisure time.

Novelty seeking is a critical component of travel motivation, acting as the polar opposite of familiarity. It is frequently defined as the degree to which one's current perception differs from one's previous experiences. Additionally, groups with disparate motives for participating in leisure activities perceive leisure constraints differently (Xu and Jiang, 2014). Cheon et al. (2005) also confirmed the effect of family travel motivation on the perception of leisure constraints. Involvement is occasionally viewed as a factor that is closely related to motivation. For instance, the term “leisure involvement” is frequently used to refer to “arousal or interest in a recreational activity or associated product” (Havitz and Dimanche, 1997). In Lee and Scott's (2009) study, involvement was used as a proxy for motivation. Their study investigated and confirmed the effect of leisure involvement on leisure constraints negotiation.

As such, this study will also examine differences in perceptions of leisure constraints among groups with varying demographic characteristics (gender, age, education, and income) and participant behavior characteristics (novelty seeking and involvement).

## Methodology

### Research Design

This study is divided into two parts. In study 1, in the context of a pandemic, the connotation of leisure constraints perceptions was investigated. The differences in respondents' perceptions of leisure constraints are being investigated in study2.

Study 1 adapted and implemented the strategy for scale development proposed by Churchill (1979) and Tsaur et al. (2010). The research procedure is divided into three stages: item generation, item purification, and scale confirmation.

#### Item Generation

The first stage of study 1 is to conduct semi-structured interviews in order to develop an item bank describing how people perceive leisure constraints in a pandemic context. The interview questions delve into great detail about the following three topics. To begin, a summary of the interviewee's demographic and leisure behavior characteristics is compiled. Second, what differences in respondents' leisure behavior and what constraining factors do they encounter during the Covid-19 pandemic, in comparison to the previous normal state? Thirdly, in the event of a Covid-19 pandemic, will the interviewee consider participating in local or international festivals? If not, why not? A snowball sampling method is used to select interview subjects for this study. This research began with interviews with people who had attended the Macau Light Festival in the previous two years. Additional tourists with comparable experiences were then identified as interview subjects as a result of their referrals. Finally, we identified and interviewed 46 interviewees. Please refer to the Appendix for basic information on the study's 46 interviewees.

Additionally, five experts were consulted regarding the revision of the items to ensure their content validity. Expert opinions are classified into three categories: item retention, item deletion, and expression modification. If two or more experts concur that an item should be deleted or modified, the researchers will do so. The appendix contains biographies of the experts who contributed to this study.

#### Item Purification

The second step in Study 1 is to process the data collected in the initial round of surveys using reliability analysis and exploratory factor analysis (EFA) in order to achieve item purification.

#### Confirmation of the Measurement Scale

The third stage of study 1 is to collect additional data in order to assess its reliability, validity, and to confirm the measurement of leisure constraints caused by the Covid-19 pandemic.

**Figure 1:**
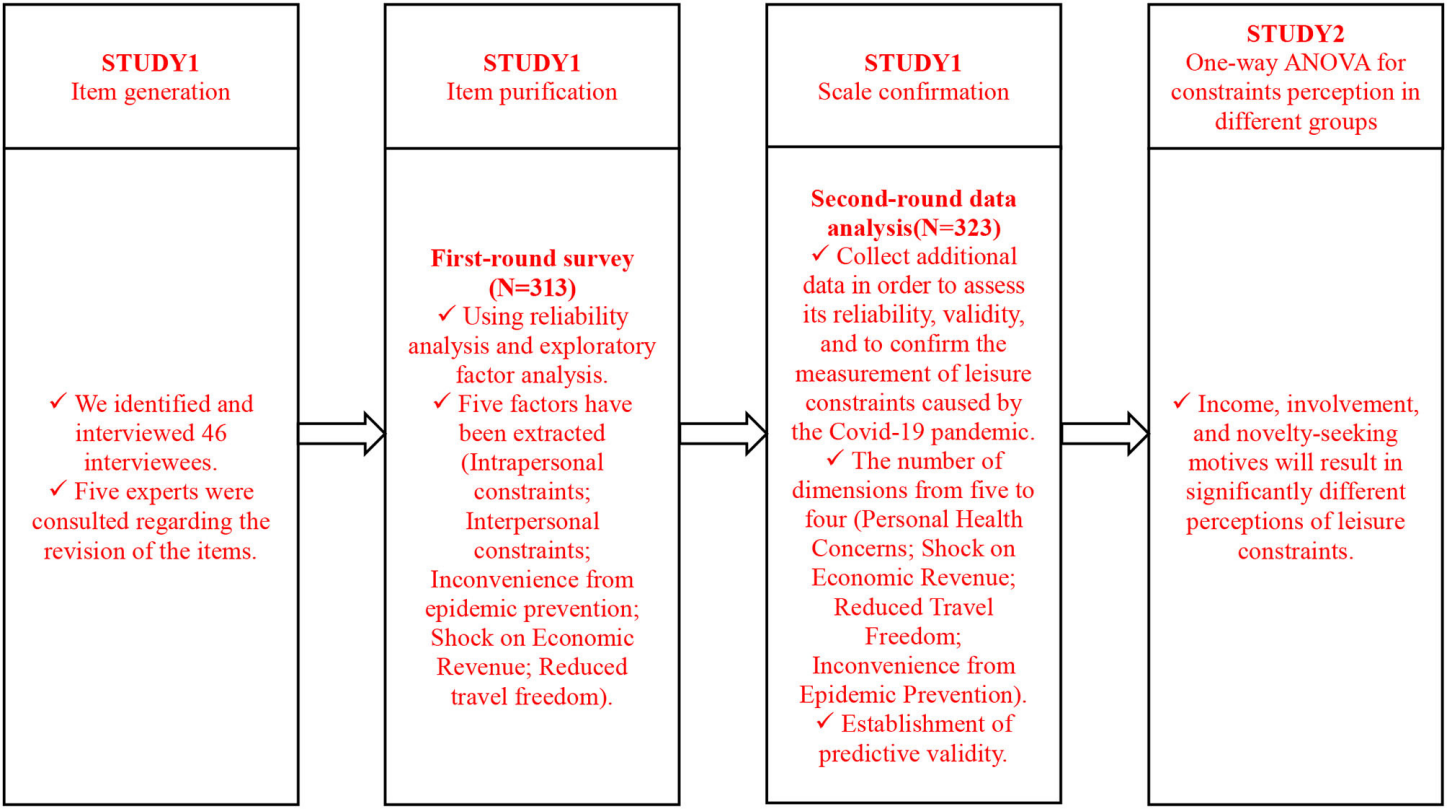
Research process.

Study 2 examines differences in perceptions of leisure constraints between different pandemic-affected groups using one-way ANOVA and paired sample *t*-tests. To illustrate the research process more clearly, Figure 1 depicts the relevant research process.

### Research Sites

Festivals are a type of recreation. As a result, the study's research context was chosen as the 2020 Macau Light Festival, which has been held in Macau for six years. The festival was originally scheduled to begin in December 2020, but was moved to October this year in order to boost Macau's tourism economy and attract tourists. The subjects of this study are tourists over the age of 18 who are aware of the Macau Light Festival.

### The Process of Data Collection

#### The First Round of Questionnaire Collection

The initial round of surveys took place between September 1 and 20, 2020. Macau only recently announced its intention to host the event in October during the time period specified above. The research team conducted the survey in Macau and Zhuhai, a city adjacent to Macau. Zhuhai survey sites are located in Gongbei port areas frequented by tourists en route to Macau. The most frequently visited areas in Macau, including Senado Square, Tap Seac Square, Nam Van Lake, and Ferrira Amaral Square, were surveyed. 350 questionnaires were distributed, and 313 responses were valid.

Regarding the respondents' personal information, there were 128 male respondents, accounting for 40.89%, and 185 female respondents, accounting for 59.11%. The primary age group is 25–34 years old (189 people), which accounts for ~60.38% of all respondents; 35–44 years old (95 people) accounts for ~30.35%. A total of 126 respondents (40.26%) had a Bachelor's degree, while 136 respondents (43.45%) had a Graduate degree or higher. Monthly incomes of the major groups are concentrated above 10,000 patacas (135 people), accounting for 43.13% of all respondents.

#### The Second Round of Questionnaire Collection

Between September 26 and October 30, 2020, the second round of the survey was conducted. The Macau Light Festival officially began during this period time. The second wave of the questionnaire survey is conducted in the same location as the first. 330 questionnaires have been distributed, and 323 valid questionnaires have been returned. There were 102 male respondents and 219 female respondents. The predominant age group (123 people) was 25–34 years old, accounting for ~38.3% of the total. A total of 176 respondents have a bachelor's degree, accounting for ~54.8% of all respondents. The monthly income-wise, respondents were more concentrated in the MOP 12,001–20,000 (55 people) range, accounting for ~17.1% of the total, followed by 54 people earning MOP 30,000 or more, accounting for 16.8%.

### Data Analysis

SPSS 24 and AMOS 23 were used to analyze data in this study. After obtaining items from interviews and purifying them with expert comments, this research uses SPSS 24 to conduct an exploratory factor analysis on the data collected in the first round of surveys in order to ascertain the internal structure of the perception of leisure constraints. Following that, this study used Amos 23 to conduct a confirmative factor analysis (CFA) on the data collected in the second round in order to ascertain the validity of the leisure constraint measurement and the structure and meaning of the latent variables. Simultaneously, based on the results of CFA, the measurement of perceived leisure constraints was modified and optimized, and the relationship between perceived leisure constraints and intention to participate in leisure activities was detected using AMOS 23 to Simultaneously, the measurement of perceived leisure constraints was modified and optimized based on the results of CFA, and the relationship between perceived leisure constraints and intention to participate in leisure activities was detected using AMOS 23 to determine the measurement's predictive validity. After completing the preceding series of tests, this study examined differences in perceptions of leisure constraints between groups with varying characteristics using the mean comparison method (Kim and Millsap, 2014) in SPSS 24.

## Findings

### Research Findings in Study 1

#### Item Generation

All 46 interviews were recorded and transcribed verbatim throughout the process. Additionally, open coding, axial coding, and selective coding modes are used to encode the interview transcription (Kassarjian, 1977). The three researchers coded the interview materials independently and came to an agreement following repeated reading and discussion.

The final coding structure formed in this study consisted of intrapersonal constraints (including six axial coding, 114 nodes), interpersonal constraints (including two axial coding, 22 nodes), and structural constraints (including nine axial coding, 192 nodes). Using the coding process described above, this study gathered 46 items on leisure constraints in the context of a pandemic. According to expert opinion, 17 items were removed from the item bank and 29 were retained in this study, including ten items for intrapersonal constraints, seven items for interpersonal constraints, and twelve items for structural constraints, which comprised the questionnaire for the initial round of the survey.

#### Results of the First-Round Data Analysis

The item analysis results indicate that all items are statistically significant at the 0.000 level, that their correlations are >0.3, and that the overall correlation of all items is >0.5. Additionally, this study examines the consistency and reliability of the items obtained in the three original dimensions. The results indicate that the Cronbach's alpha value for the original three dimensions of the question items is >0.7, indicating that they have a high degree of internal consistency (Hair et al., 2010). As a result, all 29 question items are retained. The exploratory factor analysis (EFA) revealed a Kaiser-Meyer-Olkin (KMO) value of 0.814 and a statistically significant Bartlett sphericity test of 0.000, indicating that these data are suitable for further exploratory analysis. Principal component analysis was performed using the maximum rotation variance method in the exploratory analysis, and items with an eigenvalue >1 or a factor loading >0.5 were retained (Hair et al., 2010).

The criteria outlined above were used to test 29 items in their original three dimensions repeatedly. Finally, eight items were deleted, leaving twenty-one items. At this point, five factors have been extracted, and the cumulative total variance is 72.485 % (Please see the Table 1 for details).

**Table 1:** Results of exploratory factor analysis (EFA) on data from the first round survey.

**Constructs/Items**	**Factor loading**	**Eigenvalue**	**Total variance**	**Interpretation variance**	**Alpha**
Intrapersonal constraints		6.693	19.518	19.518	0.904
Worried about the crowded participants	0.795				
Worried about the risk of the epidemic in the event venue	0.834				
Worried about being infected	0.843				
Worried about the risks that tourists will bring	0.744				
Worried about the risk of the epidemic in the destination	0.722				
Worried about the possible risk of public transportation	0.744				
Interpersonal constraints		2.779	36.698	17.179	0.894
Possible companions, do not want to participate	0.891				
My family worried that I will be infected	0.728				
My families worry that once an epidemic erupts, it will affect the family	0.850				
Possible companions are disturbed by procedures for entering and leaving public places	0.841				
Experience projects have been canceled	0.636				
Inconvenience from epidemic prevention		2.226	49.169	12.471	0.815
Entry and exit destination during the outbreak are disturbing	0.806				
Epidemic prevention practices for entering public places are disturbing	0.798				
The appointment of the nucleic acid test will influence my schedule	0.763				
The epidemic prevention and management procedures make me uncomfortable	0.740				
Shock on economic revenue		1.917	61.503	12.334	0.892
The epidemic affected my income	0.895				
The industry I work in has been hit by the epidemic	0.879				
My job prospects were impacted by the outbreak.	0.892				
Reduced travel freedom		1.607	72.487	10.982	0.828
I am not permitted to leave the local community	0.811				
Some cities are restricting me from entering	0.833				
At the moment, I cannot enter and exit a destination freely	0.910				
Kaisere Meyere Olkin measure of sampling adequacy					0.814
Barlett's test of sphericity (significance level)					0.000

#### Results of the Second-Round Data Analysis

Following the CFA analysis, it was determined that several indicators within the measurement model did not meet the requirements. The Modification Index was used to optimize and modify the measurement model, resulting in a revised measurement model. The revised measurement model retains only 12 items and reduces the dimension count from five to four. These constraints are Personal Health Concerns (PHC), which include four items such as “Worried about crowded participants;” “Worried about infection,” and so on. Shock on Economic Revenue (SER) consists primarily of two components: “The industry in which I work has been impacted by the outbreak;” and “My job prospects have been impacted by the outbreak.” Reduced Travel Freedom (RTF), which includes three items such as “I am not permitted to leave my immediate community;” “Some cities restrict my entry,” and so on. The fourth dimension is Inconvenience from Epidemic Prevention (IEP), which includes two items: “Entry and exit destinations are troublesome during an outbreak;” and “Epidemic prevention practices for entering public places are troublesome.”

CFA on the modified measurement model revealed a Chi-square value of 166.243 (df = 84, *p* < 0.000) and a chi-square/df ratio of 1.979, which was <3 and thus met the criteria recommended by Kline (2015) and Hair et al. (2010). Table 2 contains additional indicators of model fitness. All model fit indicators were found to be acceptable (Lin et al., 2017).

**Table 2:** Results of the model fit measures.

**Index**	**Chi-square**	**df**	**Chi-square/df**	**GFI**	**AGFI**	**RMSEA**	**NFI**	**RFI**	**CFI**	**IFI**
Four factors original	600.346	164	3.661	0.839	0.794	0.091	0.847	0.822	0.883	0.884
Four factors modified	166.243	84	1.979	0.938	0.911	0.055	0.931	0.913	0.964	0.964
Second-Order	175.569	86	2.042	0.934	0.908	0.057	0.927	0.911	0.961	0.961
Predictive	201.624	99	2.037	0.929	0.903	0.057	0.924	0.908	0.959	0.960
Fitted value	–	–	<3	>0.9	>0.9	<0.05	>0.9	>0.9	>0.9	>0.9

To assess internal consistency, the CR and AVE values of each latent variable were examined further in this study. The results indicate that the CR values of the four latent variables ranged between 0.8 and 0.9, exceeding Hair et al.'s (2010) recommended standard of 0.7. The AVE values of the four latent variables were all >0.5, which was consistent with Hair et al.'s (2010) recommendation. Please refer to Table 3 for specific information.

**Table 3:** Results of the CFA (*n* = 323).

**Constructs/Items**	**Estimate**	**T-Value**	**CR**	**AVE**
**Personal health concerns (PHC)**			0.882	0.600
Worried about the crowded participants	0.805			
Worried about being infected	0.822	16.888		
Worried about the risks that tourists will bring	0.742	14.822		
Possible companions, do not want to participate	0.815	16.681		
**Shock on economic revenue (SER)**			0.876	0.702
The industry I work in has been hit by the epidemic	0.912	20.713		
My job prospects were impacted by the outbreak.	0.756	16.073		
**Reduced travel freedom (RTF)**			0.880	0.648
I am not permitted to leave the local community	0.834			
Some cities are restricting me from entering	0.797	16.968		
At the moment, I cannot enter and exit a destination freely	0.807	17.279		
**Inconvenience from epidemic prevention (IEP)**			0.852	0.538
Entry and exit destination during the outbreak are disturbing	0.714			
Epidemic prevention practices for entering public places are disturbing	0.635	10.954		
The appointment of the nucleic acid test will influence my schedule	0.788	13.436		

*PHC, Personal health concerns; SER, Shock on economic revenue; RTF, Reduced travel freedom; IEP, Inconvenience from epidemic prevention*.

In Table 4, the results show that there are no problems with the construct or discriminant validity in any of the dimensions.

**Table 4:** Discriminative validity.

	**PHC**	**SER**	**RTF**	**IEP**
PHC	**0.774**			
SER	0.268^***^	**0.838**		
RTF	0.378^***^	0.184^**^	**0.805**	
IEP	0.215^**^	0.195^**^	0.470^***^	**0.733**

PHC, Personal health concerns; SER, Shock on economic revenue; RTF, Reduced travel freedom; IEP, Inconvenience from epidemic prevention. Square root of AVE on the diagonal axes in bold. Significant at **p < 0.01,

*** *p < 0.001*.

#### Predictive Validity

**Figure 2:**
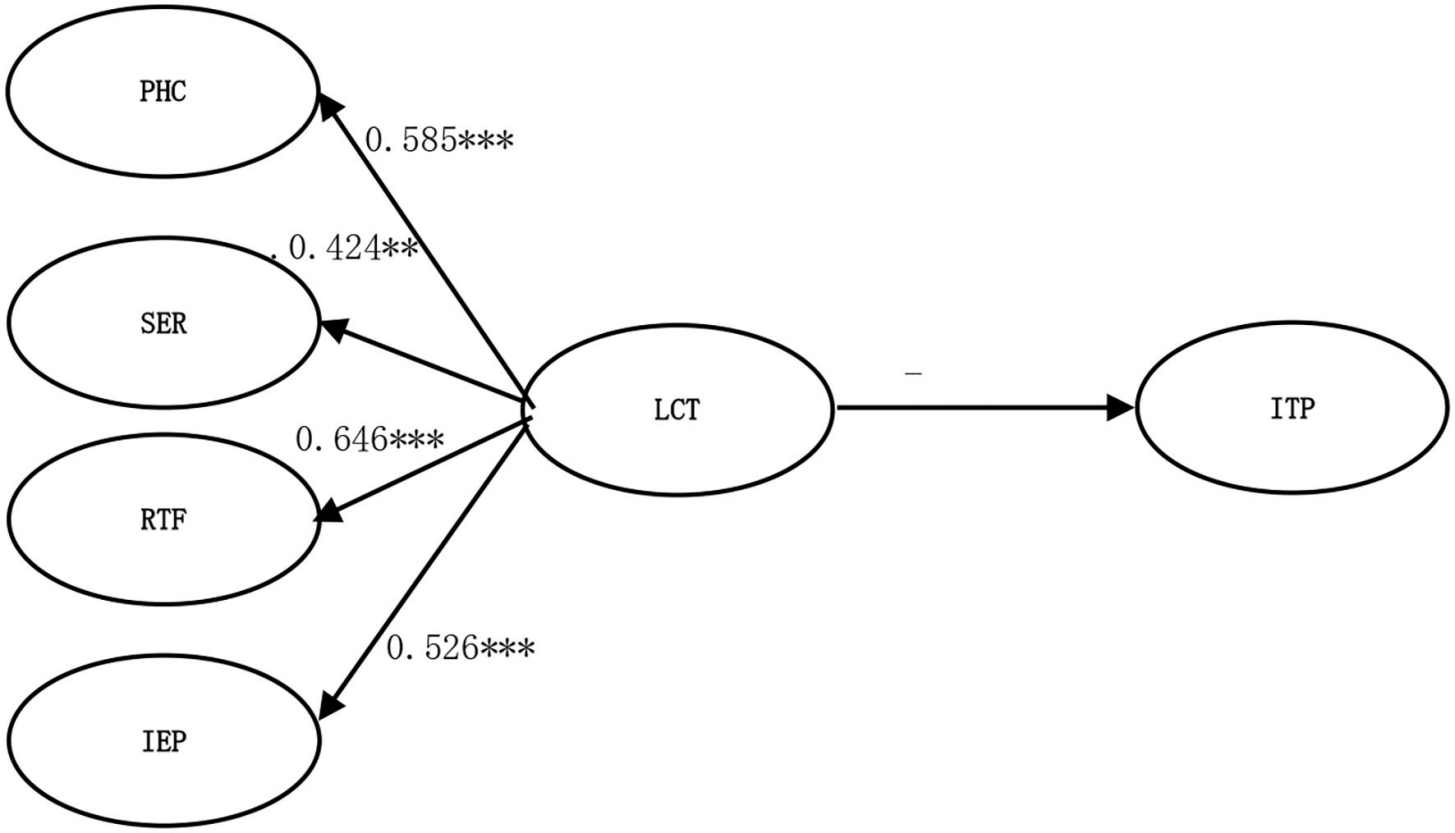
Predictive validity analysis. PHC, Personal health concerns; SER, Shock on economic revenue; RTF, Reduced travel freedom; IEP, Inconvenience from epidemic prevention; LCT, Leisure constraints; ITP, Intention to participate. Significant at **p < 0.01, ***p < 0.001.

The present study employs a structural equation model (SEM) to assess the measurement's predictive validity by examining the influence of leisure constraints on participation intention. The results indicate that model fit generally meets stringent criteria, and the path coefficients for the four leisure constraint constructs are as follows: PHC = 0.585, SER = 0.424, RTF = 0.646, and IEP = 0.526. Leisure constraints have a negative correlation with behavioral intentions, with a coefficient of−0.288, and all indicators reached a level of significance (*p* = 0.000), as illustrated in Figure 2.

### Research Findings in Study 2

The chi-square test demonstrates that participants' income, involvement, and novelty-seeking motivations all influence their perceptions of leisure constraints significantly.

To examine the differences in respondents' perceptions of constraints associated with festival and leisure activities during the pandemic, this study first calculated the average values for the four dimensions of perceived leisure constraints using the results of the preceding analysis, and then conducted one-way ANOVA tests to determine the difference. Prior to conducting the ANOVA tests, the Levene statistic was used to assess the homogeneity of the variance assumptions. The findings indicate that the hypothesis of homogeneity of variance was violated in terms of novelty seeking (*P* > 0.05), festival involvement (*P* > 0.05), and monthly income (*P* > 0.05). The ANOVA test results indicate that there are significant differences in perceptions of constraints between groups with varying degrees of novelty-seeking motivation, monthly income, and festival involvement. For more information, see Table 5.

**Table 5:** One-Way ANOVA for constraints perception in different groups.

**Source**		**Sum of squares**	**df**	**Mean square**	***F***	**Sig**.
IEP/novelty	Between groups	23.583	6	3.930	4.164	0.000
	Within groups	296.417	314	0.944		
	Total	320.000	320			
PHC/involvement	Between groups	28.400	2	14.200	15.486	0.000
	Within groups	291.600	318	0.917		
	Total	320.000	320			
IEP/involvement	Between groups	6.665	2	3.333	3.382	0.035
	Within groups	313.335	318	0.985		
	Total	320.000	320			
SER/involvement	Between groups	9.496	2	4.748	4.863	0.008
	Within groups	310.504	318	0.976		
	Total	320.000	320			
SER/salary	Between groups	26.110	7	3.730	3.973	0.000
	Within groups	293.890	313	0.939		
	Total	320.000	320			

*PHC, Personal health concerns; SER, Shock on economic revenue; RTF, Reduced travel freedom; IEP, Inconvenience from epidemic prevention*.

As a *post-hoc* analysis, the Scheffe's method was used, and the results are shown in Table 6.

**Table 6:** *Post-hoc* comparison between different groups for constraints perception.

**Comparison group**	**Mean difference**	**Sig**.
Novelty = 6/1 (IEP)	1.21	0.002
Novelty = 7/1 (IEP)	1.09	0.019
Salary = 4/1(SER)	−0.768	0.037
Involvement high/medium (PHC)	0.424	0.001
Involvement high/low (PHC)	0.821	0.000
Involvement high/medium (IEP)	0.314	0.032
Involvement high/low (SER)	0.457	0.013

*PHC, Personal health concerns; SER, Shock on economic revenue; RTF, Reduced travel freedom; IEP, Inconvenience from epidemic prevention*.

The findings indicate that groups with varying degrees of novelty-seeking motivation exhibit significant differences in IEP perception (*P* < 0.05), with an *F*-value of 4.164. *Post-hoc* comparisons reveal that respondents with novelty-seeking = 6 have a more favorable perception of IEPs than respondents with novelty-seeking = 1, and respondents with novelty-seeking = 7 have a more favorable perception of IEPs. It is more significant than the respondent group with novelty-seeking = 1.

Additionally, there are significant differences in respondents' perceptions of SER based on their monthly income level (*P* < 0.05). *Post-hoc* analysis reveals that respondents with monthly incomes less than MOP3,000 have significantly higher perceptions of SER than those with monthly incomes between MOP8,001 and MOP12,000. The F value for this difference is 3.973.

Respondents with varying degrees of festival involvement had significantly different perceptions of PHC, IEP, and SER, with *P* < 0.05 and *F*-values of 15.486, 3.382, and 4.836, respectively. More precisely, respondents with a higher level of festival involvement have significantly more favorable perceptions of PHC, IEP, and SER than respondents with a lower level of festival involvement.

## Discussions

### Theoretical Implication

The primary goal of this study is to ascertain participants' perceptions of the leisure constraints associated with festival attendance in the context of the Covid-19 Pandemic. Finally, 12 items were obtained, which can be classified into four dimensions: Personal Health Concerns (PHC), Shock on Economic Revenue (SER), Travel Freedom Restrictions (RTF), and Inconvenience from Epidemic Prevention (IEP).

By comparing the findings of this study to those of previous research on leisure constraints, it is clear that this study combines the two dimensions of intra-personal and inter-personal constraints identified in previous research. Furthermore, the vast majority of interpersonal constraints are removed. Given that this study was conducted during the Covid 19 pandemic, prior research on this scenario established that the most immediate danger is to one's personal health (Neuburger and Egger, 2021). Simultaneously, maintaining social distance has become the new normal as a result of the pandemic (Bae and Chang, 2021; Neuburger and Egger, 2021). As a result, interpersonal interaction's influence on consumer behavior is diminished. At the moment, the impact of the Covid-19 pandemic on people's perceptions of the constraints associated with recreational activities is more reasonable in terms of intra-personal constraints.

In terms of the composition of the respondents' perceived leisure constraints, the RTF imposes the most significant constraints. RTF has a path coefficient of 0.646 in the predictive validity analysis model, while PHC has a path coefficient of 0.585. This finding is intriguing because, despite the fact that the research was conducted during a pandemic, respondents did not place a high premium on their personal health. Individuals place a premium on what they believe is significant (Tempesta et al., 2010). Given the primacy of physiological and safety needs (Maslow, 1958), it is reasonable to assume that respondents will place a premium on their perceptions of health risks during a pandemic.

The study's unique circumstances may account for the aforementioned unexpected findings. Due to the fact that Maslow's hierarchy of needs is a broad description of human needs, whereas this research is focused on leisure behavior. Leisure is a behavior that occurs only after basic needs are met from a hierarchical perspective. Respondents may be more focused on leisure's fundamental connotation, which is a sense of freedom (Næss, 2006; Carr, 2017). Simultaneously, accessibility is a necessary condition for participation in public recreational activities (Næss, 2006). By 2020, 90% of countries and regions affected by the pandemic have imposed travel restrictions (Gössling et al., 2020). At this point, respondents may perceive obvious constraints on their accessibility and capacity for freedom. As a result, respondents view RTF as the most significant constraint on their leisure time.

PHC is ranked second in this study's list of leisure constraints. Earlier scholars, such as Funk et al. (2009), asserted that perceptions of leisure constraints are negatively correlated with willingness to participate, with intra-personal constraints having the greatest influence. Another example is the study by Gilbert and Hudson (2000), which demonstrates that intra-personal constraints have the greatest path coefficient of influence on behavioral intent, while structural constraints have the smallest path coefficient. COVID-19 is more contagious than previous strains of SARS and MERS (Bae and Chang, 2021). As a result, the pandemic's trajectory will have a profound effect on individuals (Halloran et al., 2008), increasing individual perceptions of health risks (Davies, 2021; Neuburger and Egger, 2021).

Individuals' perceptions of leisure constraints vary according to their life stage and social status (Shores et al., 2007; Godbey et al., 2010). The purpose of this study was to compare respondents' perceptions of leisure constraints during the pandemic. According to Xu and Jiang (2014), perceptions of leisure constraints are related to personal leisure motivation, and festival attendees exhibit a strong desire for novelty (Cheng et al., 2015). Previous research has established a positive correlation between leisure motivation and perceived constraints, indicating that the more intense the motivation for leisure activities, the more obvious the perceived constraints (Zhang, 2010). The findings of this study corroborate the aforementioned scholars' positions. Along with novelty seeking motivation, this study examines the relationship between perceived leisure constraints and level of involvement. While some scholars regard involvement as a critical part of leisure constraints (Nuijten et al., 2016), involvement is synonymous with motivation in this study. According to the study's findings, groups with a higher level of festival involvement will face more severe leisure constraints than groups with a lower level of involvement. This also demonstrates that motivation and the perception of constraints are two concurrent internal psychological processes. A strong internal motivation will also result in a strong awareness of leisure constraints (Zhang, 2010).

Additionally, the study's findings indicate that the lower the monthly income, the greater the constraints on leisure imposed by economic shocks. Previously conducted research has espoused similar views. For example, Chang et al. (2011) and Boo et al. (2014) both stated that low-income individuals face greater intrapersonal, interpersonal, and structural constraints. Prior to engaging in leisure activities, people must be free of work and daily chores (Voss, 1967); for groups with lower income levels, when they perceive their income is being reduced, their time allocation between work and leisure becomes more cautious. The pandemic of COVID-19 has thrown the global economy into unprecedented turmoil (Davies, 2021), making it more difficult for low-income individuals to work less and engage in leisure activities.

### Management Implications

This study is instructive for festival organizers and managers operating in the post-pandemic era and other times of crisis. When local governments consider festivals as a means of promoting economic development in the post-pandemic phase, they should prioritize analyzing potential participants' perceptions of leisure constraints. They can only increase festival attendance by reducing their perceived constraints on leisure. While preventing epidemics is critical in the event of a pandemic, organizers should also prioritize creating a recreational environment. For instance, festival organizers can provide real-time information about social distance (such as the number of attendees) to assist participants in alleviating their personal health concerns (Zheng et al., 2021).

Additionally, participants should take an active role in vaccination to boost their confidence and reduce their risk of infection while attending festivals (Dash and Sharma, 2021). Additionally, the organizer should establish a dedicated hygiene and epidemic prevention team and require all staff to strictly wear masks to heighten and reinforce staff awareness of epidemic prevention (Kaushal and Srivastava, 2021). Organizers should specifically undermine the perception of leisure constraints among loyal participants with a strong interest in festivals and potential participants with a lower income level. According to studies, consumers are more willing to participate in small group, safe, and secure leisure activities following the pandemic (Bae and Chang, 2021). Leisure activity planning can adopt novel organizational structures that enable participants experiencing a crisis to reclaim their attention as quickly as possible (Li et al., 2020), thereby alleviating perceived constraints. For example, the original large-scale festival could be divided into a series of miniaturized theme festivals, lowering the perceived leisure constraints of potential participants.

### Limitations of Research

While this research has yielded enlightening results, the research process has the following limitations:

To begin, this research examines the leisure constraints imposed by the public health crisis through the lens of festivals. As a result, the research's conclusions have a limited range of application. The findings of the research should then be replicated for a variety of leisure activities.

Second, this study did not consider the stage of development of the health crisis. As a result, additional discussion is necessary to ascertain whether the study's findings are applicable to other stages of the crisis.

## Data Availability Statement

The original contributions presented in the study are included in the article/supplementary material, further inquiries can be directed to the corresponding author.

## Ethics Statement

Ethical review and approval was not required for the study on human participants in accordance with the local legislation and institutional requirements. The patients/participants provided their written informed consent to participate in this study.

## Author Contributions

XL and JL: research development. YX and CX: survey. XS and XL: research method. JL, XL, and YX: manuscript writing and proofreading. All authors contributed to the article and approved the submitted version.

## Conflict of Interest

The authors declare that the research was conducted in the absence of any commercial or financial relationships that could be construed as a potential conflict of interest.

## Publisher's Note

All claims expressed in this article are solely those of the authors and do not necessarily represent those of their affiliated organizations, or those of the publisher, the editors and the reviewers. Any product that may be evaluated in this article, or claim that may be made by its manufacturer, is not guaranteed or endorsed by the publisher.
